# A Rare Axonal Variant of Guillain-Barré Syndrome following Elective Spinal Surgery

**DOI:** 10.1155/2018/2384969

**Published:** 2018-08-07

**Authors:** Jessica R. Dowling, Thomas J. Dowling

**Affiliations:** ^1^Charles E. Schmidt College of Medicine at Florida Atlantic University, 777 Glades Road, Boca Raton, FL 33431, USA; ^2^Long Island Spine Specialists PC, 763 Larkfield Road, Commack NY 11725, USA; ^3^St. Catherine of Sienna Medical Center, 50 New York 25A, Smithtown, NY 11787, USA

## Abstract

Acute motor and sensory axonal neuropathy (AMSAN) is a rare axonal variant of Guillain-Barré syndrome. AMSAN is considered the most severe form of GBS, known for its rapid onset of severe symptoms, and often leading to quadriparesis within 7 days of initial symptom onset. We present a case of a middle-aged Caucasian female who developed AMSAN 2 weeks following an elective spinal surgery. Although rare, GBS has been reported as a complication of surgery. GBS classically presents as ascending motor weakness starting in the lower extremities following a gastrointestinal or upper respiratory tract infection. This patient's GBS manifested slightly differently, with both sensory and motor symptoms of her thoracic region and lower extremities, with no preceding history indicative of infection. To the authors' knowledge, this is the first reported case of AMSAN following spinal surgery. Because of its risk of significant morbidity and mortality, as well as similar presentation to more common spinal postoperative complications, GBS should always be included in the differential diagnosis whenever motor or sensory weakness is observed after spinal surgery.

## 1. Introduction

We present a case of acute motor and sensory axonal neuropathy (AMSAN), a rare axonal variant of Guillain-Barré syndrome (GBS), 2 weeks following an elective spinal surgery in a middle-aged woman. To the authors' knowledge, this is the first reported case of AMSAN following spinal surgery, although postoperative GBS has been previously reported in the literature. It is imperative that orthopedic surgeons, especially spinal surgeons, keep GBS on the differential following surgery as it may present very similarly to other, more common postoperative complications.

## 2. Case: Presentation

### 2.1. Presentation

A 53-year-old white female presented to outpatient orthopedics with a chief complaint of sharp lumbar back pain radiating down her right leg for 2 weeks. The pain was a 7/10 on the visual analogue scale. Review of symptoms was positive for fatigue and negative for bowel or bladder dysfunction. Pertinent physical exam findings included the following: no tension signs, right antalgic gait, right paraspinal tenderness, and normal lumbar spine range of motion. The neurologic exam was normal except for an absent right patellar reflex. The patient had previously underwent multiple lumbar spine surgeries related to a work injury, including L4-L5 right endoscopic discectomy, L3-S1 laminectomies and posterolateral instrumental fusion, L2-L3 lateral left interbody fusion with posterior instrumentation, and correction of L1-L2 segmental kyphosis with extension of fusion to T10 with T10-L5 posterior segmental instrumentation. X-rays and MRIs were obtained at this time, showing instability and spinal stenosis at L1-2. This was above her prior fusion level.

### 2.2. First Operation and Postoperative Course

The patient elected to have a L1-2 laminectomy with removal of all prior posterior spinal instrumentation and extension of the posterior fusion from L2-T10 with instrumentation placed posterior from T10-L5. She had no acute complications following surgery and was discharged on postoperative day 5.

**Figure 1:**
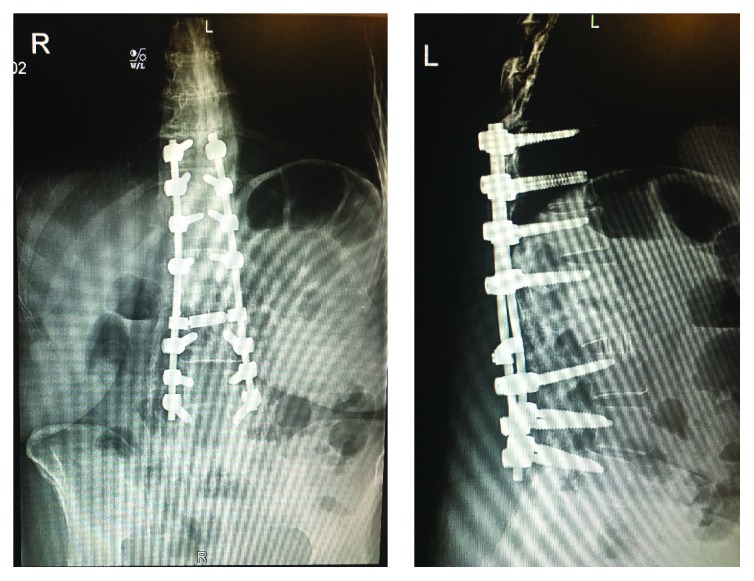
AP and lateral X-ray views at 2-week postoperative visit show T10-L5 posterior segmental instrumentation with evidence of interbody fusion with cage at L2-L3. Evidence of prior fusion at L2-S1 noted. New laminectomy at L1-L2 with prior lumbar decompression noted distally.

At the patient's 2-week postoperative visit, she reported a 2-day history of acute onset excruciating pain over her lower thoracic region with radiation throughout her ribs and lower back. She also had difficulty walking due to diffuse, ascending weakness, pain, numbness, and tingling in her legs bilaterally, without bowel or bladder dysfunction. The patient reported that she was doing well until the onset of these symptoms and denied any preceding fevers or signs of infection. On physical exam, she was afebrile and her wound was healing well. Neurologic exam revealed varying degrees of muscle weakness in all lower extremity groups with diffuse hyposensitivity to touch over dermatomes L3-S1. There was trace patellar reflexes and absent Achilles reflex bilaterally. She received an immediate thoracic and lumbar MRI with and without contrast. MRI showed no significant cord compression or epidural hematoma or abscess, although there was difficulty interpreting the studies due to extensive spinal hardware. Further imaging was obscured due to her extensive spinal instrumentation from prior surgeries (Figure 1).

### 2.3. Second Operation and Postoperative Course

**Figure 2:**
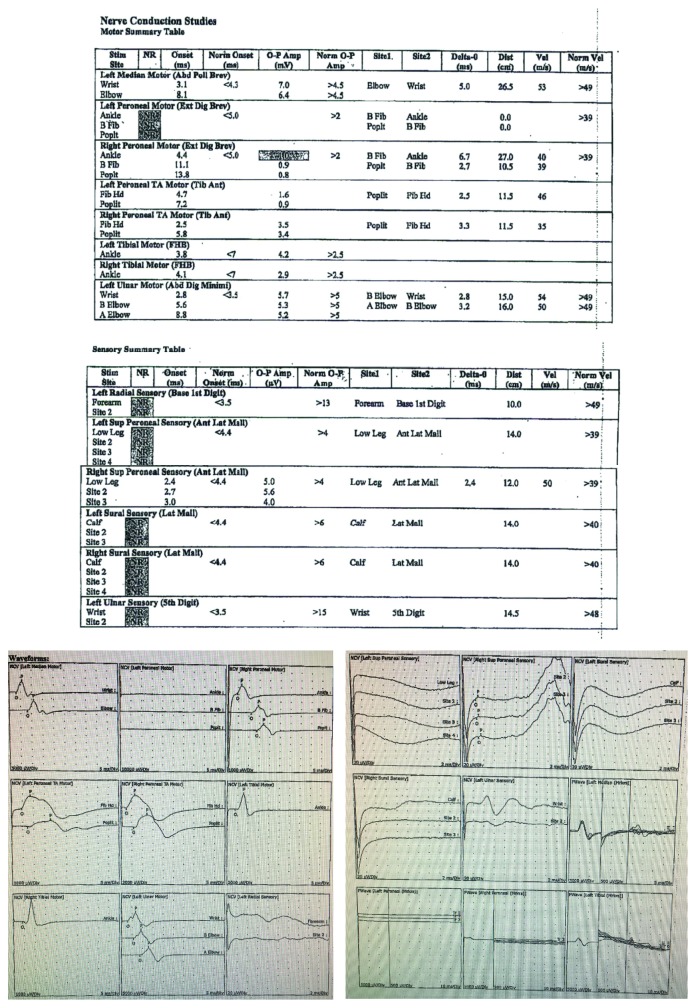
Summary table of the patient's motor and sensory findings on electrodiagnostic examination and EMG waveforms, respectively. The above examination shows no response of left peroneal motor nerve, left radial sensory nerve, left superior peroneal sensory nerve, left sural sensory nerve, right sural sensory nerve, and left ulnar sensory nerve. There is reduced amplitude of right peroneal motor nerve. Evaluation of left peroneal and right peroneal F-wave showed no response, and left tibial F-wave showed prolonged latency (not pictured above). Left and right tibial H-reflexes were abnormal. EMG needle evaluation of the right anterior tibialis showed no polyphasic potentials and reduced recruitment. Left anterior tibialis showed no polyphasic potentials. Left iliopsias showed no polyphasic potentials and reduced recruitment.

The patient returned to the operating room 1 week later for exploration of the spine due to the inconclusive imaging and worrisome symptoms and physical exam findings. Cross-link instrumentation was removed at this time, and her laminectomy at L1 was revised. There was no evidence of postoperative hematoma or spinal compression noted intraoperatively. Neurology was consulted, and postoperative nerve conduction testing and electromyography (EMG) were performed in order to further elucidate the cause of her symptoms (Figure 2). EMG and nerve conduction studies showed generalized nonlength-dependent sensory greater than motor polyneuropathy with mostly axonal features. Given the acute onset of her symptoms and lack of significant demyelinating features on nerve conduction studies, the findings were indicative of AMSAN, which is an axonal variant of GBS.

The patient was started on intravenous immunoglobulin (IVIG) therapy as well as antiganglioside antibodies. During her first month of treatment, she experienced partial neurologic recovery and then relapsed back to her baseline symptoms at 6-week postoperative. She is currently in in-patient rehabilitation being treated with physical therapy, IVIG, and antiganglioside antibodies with minimal neurologic recovery at this time.

## 3. Discussion

GBS is an acute peripheral neuropathy caused by an autoimmune response against the myelin of peripheral nerves. This syndrome is rare within the United States, with an incidence of 1-2 cases per 100,000 persons [1]. GBS has a reported mortality rate of 3–15% and necessitates mechanical ventilation in 25% of its patients [2]. GBS may be diagnosed based on clinical observations, cerebrospinal fluid (CSF) analysis, and/or nerve conduction studies. A CSF analysis was not obtained due to the high likelihood of an unsuccessful and complicated spinal tap due to the patient's multioperated thoracolumbar spine with vast instrumentation. A cisternal puncture would have been required to obtain a satisfactory specimen. Additionally, there was concern over CSF contamination due to the patient's recent spinal surgery [2].

Once diagnosed, GBS can be treated with supportive care as well as plasma exchange and/or IVIG in attempt to neutralize the circulating antigen-immune complexes. Treatment can help accelerate the healing process and prevent catastrophic morbidities; however, the typical recovery from GBS can take up to 2 years, with lasting neurologic effects in 20% of patients [3].

GBS classically presents after a gastrointestinal or upper respiratory tract infection with ascending motor weakness starting in the lower extremities with associated autonomic dysfunction, such as cardiac arrhythmias and urinary retention. The most feared complication of this syndrome is motor weakness that ascends to the diaphragm, causing respiratory failure and possible death. This patient's GBS manifested slightly differently, with pain and sensory symptoms of her thoracic region and lower extremities, in addition to the classic motor deficits, with no history of preceding infection.

AMSAN is a rare and severe subtype of GBS, accounting for only 3–5% of all cases of GBS in Western countries. Electrophysiology studies may differentiate this subtype from the other forms of GBS. AMSAN usually presents with severe symptoms over a short period of time, and patients often experience a prolonged and incomplete recovery compared to other forms of GBS, as seen in this patient [4, 5].

GBS has a reported postoperative incidence of 4.1 cases per 100,000 patients for the general population of the United States and an incidence of 1 case per 2000 patients following spinal surgery [2]. In most cases, GBS develops 1–3 weeks following surgery [6]. The mechanism behind the increased risk of GBS following surgery has not yet been fully elucidated. However, the leading hypothesis in the literature is that the release of antigens intraoperatively may lead to subsequent autoimmunization of these antigens. Additionally, surgery has been shown to alter the balance of the immune system, which may act as a trigger for the development of GBS or predispose patients to subclinical infections that may lead to GBS [2].

GBS following spinal surgery poses a unique diagnostic challenge as it presents similarly to other, more common postoperative spine complications such as hemorrhage, infection, vascular injury/ischemia, and acute spinal cord or nerve root compression. Paresthesia, pain, and paralysis, typically symptoms of GBS, may also be presenting signs of these other more common postoperative complications. GBS may be distinguished from these other conditions by imaging and surgical exploration. More common postoperative complications, such as hematoma or nerve root compression, would have positive radiologic results and be seen on surgical exploration, whereas GBS would have negative findings.

Because of GBS's significant risk of morbidity and mortality, specifically paralysis and respiratory failure, GBS should be included in the postoperative differential diagnosis whenever motor weakness, or as seen in this case, sensory symptoms are observed after surgery. This is especially important following spinal surgery, as GBS presents very similarly to other more common, postoperative spinal complications.
